# A MEDITERRANEAN DIET INTERVENTION ALTERS AGE-ASSOCIATED PHYSIOLOGY IN A NOVEL NON-HUMAN PRIMATE MODEL

**DOI:** 10.1093/geroni/igz038.264

**Published:** 2019-11-08

**Authors:** Noah Snyder-Mackler, Carol Shively, Corbin Johnson, Kristopfer Michalson, Susan Appt, Daniel Belsky, Thomas Register

**Affiliations:** 1 University of Washington, Seattle, Washington, United States; 2 Wake Forest University, Winston-Salem, North Carolina, United States; 3 Columbia University Mailman School of Public Health, New York, New York, United States

## Abstract

Diet modifications are some of the most well-established aging interventions. For decades we have known that caloric restriction can dramatically increase lifespan and healthspan in organisms ranging from yeast to primates. More recently, other dietary modifications, including varying nutrient composition, have been experimentally shown to alter healthspan and lifespan. However, limitations inherent in human trials, such as diet adherence and heterogeneity of other lifestyle factors, mitigate our ability to identify the mechanisms through which diet alters healthspan and lifespan. Here, we conducted a randomized, long-term, whole-diet manipulation in a nonhuman primate, where cynomolgus macaques consumed either a Mediterranean or Western diet for 15 months. We hypothesized that individuals fed a Western diet would exhibit accelerated rates of cellular and physiological aging relative to their Mediterranean-fed counterparts. Indeed, we found that Western diet-fed animals exhibited increases in physiological measures that also increase with age, including body weight, fasting insulin, and triglycerides. Animals eating a Mediterranean diet, on the other hand, had a more sensitive and tuned autonomic response, and reduced HPA responses to an acute stress challenge. Probing further, we found that diet strongly affected monocyte function, altering the expression of 40% of expressed genes, leading to a more proinflammatory monocyte phenotype in Western diet fed animals. Experiments are underway to explore effects of diet on other markers of biological aging. Together, these data provide the first controlled evidence that Western and Mediterranean diets can alter aging-associated function in a species with clear biological similarity and relevance to humans.

